# Impact of Starch Concentration on the Pasting and Rheological Properties of Gluten-Free Gels. Effects of Amylose Content and Thermal and Hydration Properties

**DOI:** 10.3390/foods12122281

**Published:** 2023-06-06

**Authors:** Raúl Ricardo Mauro, Antonio José Vela, Felicidad Ronda

**Affiliations:** Department of Agriculture and Forestry Engineering, Food Technology, College of Agricultural and Forestry Engineering, University of Valladolid, 34004 Palencia, Spain; raul.mauro@uva.es (R.R.M.); antoniojose.vela@uva.es (A.J.V.)

**Keywords:** innovative gluten-free foods, gluten-free gels, starch, rheological properties, thermal properties, amylose content, techno-functional properties

## Abstract

The pasting and rheological properties of starch gels from different botanical origins have been widely used to evaluate the application of these starches in pharmaceutical and food products. However, the ways in which these properties are modified by starch concentration and their dependence on amylose content and thermal and hydration properties have not been adequately established so far. An exhaustive study of the pasting and rheological properties of starch gels (maize and rice (normal and waxy in both cases), wheat, potato, and tapioca) at concentrations of 6.4, 7.8, 9.2, 10.6, and 11.9 g/100 g was performed. The results were evaluated in terms of a potential equation fit between each parameter and each gel concentration. The parameters determined for the gels at the studied concentrations were correlated with the hydration properties and thermal properties by applying principal component analysis (PCA). Wheat starch, followed by normal maize and normal rice starches, presented a greater capacity to modulate their gels’ pasting and viscoelastic properties via their concentration in water. On the contrary, the characteristics of waxy rice and maize, potato, and tapioca starches were barely modified by concentration in pasting assays, but the gels of potato and tapioca showed noticeable changes in their viscoelastic properties as functions of concentration. In the PCA plot, the non-waxy cereal samples (wheat, normal maize, and normal rice) were located close to each other. Wheat starch gels were the most dispersed on the graph, which is consistent with the high dependence on the concentration of the gel shown in most of the studied parameters. The waxy starches had close positions not too distant from those of the tapioca and potato samples and with little influence from amylose concentration. The potato and tapioca samples were close to the vectors of the crossover point in rheology and peak viscosity in their pasting properties. The knowledge gained from this work allows a better understanding of the effects of starch concentration on food formulations.

## 1. Introduction

The use of native starches is one of the most popular strategies for the development of gluten-free products. They are used in a wide variety of food products as thickeners, gelling agents, stabilizers, bulking agents, and water absorbers [1].

Rice and maize starches are the most commonly used starches in the gluten-free bakery industry [2,3], whereas tapioca and potato starches have been tested as ingredients that significantly modify the technological properties of food products [3,4,5,6]. Rice, wheat, maize, potato, and cassava starches are among the most produced starches in the world [7]. Starch mixtures and the concentrations of each material in the products could determine their behavior against structure modifications such as retrogradation due to the different interactions between the amylose and amylopectin chains of each starch [8]. Some blends, such as waxy rice starch and waxy potato starch, would improve the nutritional profile of the products in which they are applied by reducing the amount of rapidly digestible starch [9]. Functional, calorimetric, and viscoelastic tests provide information on the behavior of starches when used as raw materials. Hydration has been documented as a critical factor in many manufacturing processes [10]. Hydration properties are important in viscous foods such as soups, sauces, doughs, and baked products where good interaction between water and the food matrix is required [11].

Swelling power, amylose content, and paste viscosity provide important information to infer their potential in the development of products such as biscuits [12], whereas the water absorption index (WAI) and water solubility index (WSI) correlate well with the cooking properties and acceptability of products such as pasta [13]. In pasting studies, samples with higher amylose content generally have lower peak viscosities, higher final viscosities, and higher pasting temperatures than their counterparts with lower or no amylose content because granule swelling is more difficult due to blockage caused by leached amylose [2,14,15]. It has been observed that some foods with low amylose and with pasting profiles with high peak viscosity and low final viscosity are more resistant to retrogradation and give a more desirable texture to some pastas [16] and other specialty products such as tortillas [17], although a high level of amylose content would be beneficial to human health due to slower digestion and absorption [16]. 

Starch dispersions are subjected to combined heating and shearing effects during processing, which affect their rheology and the final product’s characteristics [18]. However, it is still necessary to study whether the variation of starch concentration in the gels is a determinant of these parameters. 

In the present work, the pasting and rheological properties of starch gels (maize and rice (normal and waxy in both cases), wheat, potato, and tapioca) at concentrations ranging from 6.4 to 11.9 g/100 g were studied. The results were evaluated in terms of the fit to a potential equation between the studied parameters and the concentrations of the gels. The parameters determined for the gels at the concentrations studied were correlated with their hydration properties (Water Absorption Capacity (WAC), Water Absorption Index (WAI), Water Solubility Index (WSI), and Swelling Power (SP)) and thermal properties by applying principal component analysis (PCA). 

## 2. Materials and Methods

### 2.1. Samples

Normal maize (C*Gel 03401), waxy maize (C*Gel 04201), wheat (C*Gel 20006), potato (C*Gel 30002), and tapioca (C*CreamGel 70001) starches were manufactured by Cargill (Cargill Inc., Minneapolis, MN, USA) and kindly donated by Brenntag Química S. A. U. (Dos Hermanas, Seville, Spain). Waxy rice (Remyline XS) and normal rice (Remy DR) starches were obtained from BENEO (BENEO GmbH, Mannheim, Germany) and kindly provided by Ferrer Alimentación (Barcelona, Spain). The starches analyzed had a purity greater than 99% on a dry basis, according to the commercial information available.

### 2.2. Moisture Content

Moisture determination was carried out by applying the AACC method 44-19.01 [19]. Briefly, a sample of 2 g was placed in a capsule of known weight, dried in an oven at 135 °C for 2 h (until minimum constant weight), and cooled to room temperature in a desiccator. The mass of the dry matter was weighed, and the percentage of moisture was calculated from the difference. Samples were measured in duplicate.

### 2.3. Amylose Determination

The amylose concentration was determined using the Amylose/Amylopectin Determination Kit (K-AMYL 06/18) from Megazyme (Wicklow, Ireland), applying the procedure of Gibson et al. (1997) [20]. As described in the kit’s instructions, the starches were completely dispersed by heating them in DMSO, and the lipids were removed by precipitating the starch with ethanol and discarding the supernatant. The precipitated sample was dissolved in a solution of acetate and salt. Amylopectin was specifically precipitated by the addition of concanavalin A (Con A) and then removed via centrifugation. Amylose was enzymatically hydrolyzed to D-glucose and analyzed using the glucose oxidase/peroxidase reagent (GOPOD). Similarly, a separate aliquot of the acetate/salt solution was hydrolyzed to D-glucose and color-measured using GOPOD for the determination of total starch using a glucose standard of 1 g/L. The amylose content was estimated as the ratio of the two GOPOD-treated solutions’ absorbances at 510 nm of the supernatant of the samples precipitated with Con A to the absorbance of the total starch determination. The results refer to dry matter. Samples were measured in duplicate.

### 2.4. Hydration Properties

The water absorption capacity (WAC) of the starches was determined by using the centrifugation method described by several authors [11,21,22,23,24]: 2 g of each starch was mixed with 20 mL of distilled water to form a dispersion at room temperature, vortexed 3 times at low speed for 30 s, and allowed to stand for 10 min between each vortexing, followed by centrifugation at 3000× *g* for 30 min. The supernatant was discarded, and the weight of the precipitate (hydrated starch weight) was recorded, from which the mass of incorporated water was calculated. The WAC value is expressed as g water/g starch. 

Water absorption index (WAI), water solubility index (WSI), and swelling power (SP) tests were performed as described by Abebe et al. (2015) [11] with slight modifications: 2 g of each starch were dispersed in 20 mL of distilled water and vortexed, then placed in a boiling water bath for 15 min, allowed to cool to room temperature for about 1 h, and centrifuged at 3000× *g* for 10 min. The supernatant was collected in capsules and dried at 110 °C for 24 h. The weight of the drained supernatant was recorded. WAI, WSI, and SP values were expressed in terms of g gel/g starch, g soluble solids/100 g starch, and g gel/g insoluble matter in starch, respectively. 

All values obtained were corrected for the moisture content of each sample and are expressed on a dry basis. Samples were measured in duplicate.

### 2.5. Thermal Properties

A differential scanning calorimeter (DSC) (DSC3, STARe System, Mettler Toledo, Switzerland) was used to evaluate the thermal transitions, including gelatinization, retrogradation, and amylose–lipid complex dissociation in starch samples using a protocol previously applied to other samples [10,25]. About 6 mg of each sample was quantitatively added to a 40 μL aluminum pan with sufficient water to reach a starch:water ratio of 30:70 *w*/*w*. Each capsule was hermetically sealed and placed in the equipment for scanning from 0 to 120 °C at a heating rate of 5 °C/min, using an empty pan as reference. Indium and zinc were used for calorimeter calibration. The enthalpy change (ΔH, J/g dry basis) and onset (To), peak (Tp), and ending (Te) temperatures were recorded for the gelatinization (G), retrogradation (R), and amylose–lipid dissociation (A-L) peaks. Endothermic transitions of retrograded starches were evaluated with a second scan conducted after storing the gelatinized samples in DSC pans for 7 days at 4 °C, following the same procedure used to evaluate gelatinization. This low storage temperature allowed faster amylopectin recrystallization, resulting in higher retrogradation enthalpies and more accurate results. At this temperature, the melting enthalpy of the recrystallized amylopectin reached a levelling-off value in about 7 days [26]. The degree of retrogradation (DR) was calculated as the quotient of the enthalpy of retrogradation and the enthalpy of gelatinization, and it is expressed as a percentage. The A-L peak was quantified from the second scan because the best conditions for complex formation occur after sample gelatinization [27]. Each sample was measured in duplicate.

### 2.6. Pasting Properties

The pasting tests were carried out using a Rapid Visco Analyzer (RVA), a Perten RVA 4500 instrument (PerkinElmer Inc., Waltham, MA, USA), following the STD1 test detailed in the official AACC method 76–21.02 [28]. Five concentration levels were applied. Amounts of starch between 2.0 and 4.0 g (14% moisture basis) were weighed and mixed with 25 g of distilled water before being loaded into the canister. The final concentration values are given in Table 1. 

Once the pasting profiles were obtained, the viscosity parameters were identified as follows [29,30,31]: peak viscosity (PV) is the maximum viscosity between the viscosity increase during heating and the viscosity drop due to granule breakage; trough viscosity (TV) is the minimum viscosity after granule collapse; breakdown viscosity (BV) is the difference between PV and TV; final viscosity (FV) is the viscosity at the end of the test, coinciding with the end of the cooling and temperature maintenance stage, in which the molecules re-associated, thereby causing a viscosity increase; setback viscosity (SB) is the difference between FV and TV; pasting temperature (PT) is the temperature at which the granules begin to swell due to water uptake; peak time (Pt) is the time at which PV appeared.

### 2.7. Rheological Properties of Gels

A dynamic oscillatory test was conducted to analyze the gels using a Kinexus Pro+ rheometer (Malvern Instruments Ltd., Malvern, UK) equipped with 40 mm parallel plates of serrated surface, working at a 1 mm gap and a constant temperature of 25 °C. Gel samples were prepared using the method described in Section 2.6. After preparation, the gel was allowed to rest on the plates for 5 min to relax before measurement. Frequency sweeps were conducted in the Linear Viscoelastic Region (LVR) at a constant strain of 1%, ranging from 1 to 10 Hz. Power law was used to model the evolution of G′, G″ and of tan (δ) versus the oscillation frequency (*F*): (1) G′ = G′_1_·F^a^, (2) G″ = G″_1_·F^b^, and (3) tan (δ) = tan (δ)_1_·F^c^. This model proved to be valid and robust, as all fits achieved correlation coefficients R^2^ > 99.9%, confirming that these viscoelastic properties increase with concentration following a potential evolution. The coefficients of the potential equation, which represent the elastic and viscous moduli as well as the loss tangent, respectively, at a frequency of 1 Hz were named G′_1_, G″_1_, and tan(δ)_1_. The exponents of the potential equation, named *a*, *b*, and *c*, quantify the dependence of the elastic and viscous moduli and the loss tangent, respectively, to the oscillation frequency. Strain sweeps were performed at 1 Hz in the range of 0.1 to 1000% except for tapioca and potato starches, where the range was extended to 2500%. The maximum stress in the LVR and the stress at the cross point (G′ = G″) were determined from strain sweeps. The end of the LVR (τ_max_) was identified as the sharp decrease in the G′ modulus, coinciding with the sudden increase in tan(δ). 

### 2.8. Statistical Analysis

Shapiro–Wilk normality tests, statistical analyses of mean, standard deviation, ANOVA with Tukey’s test of comparisons, and principal component analysis (PCA) were performed using the free software R version 4.2.2 (The R Foundation) under the IDE RStudio 2022.12.0 + 353 (Posit Software, Boston, MA, USA). The fitting of the studied parameters as a function of starch concentration to the potential equation was performed with the statistical package Statgraphics Centurion 19—Version 19.4.01 (Statgraphics Technologies Inc., The Plains, VA, USA). 

## 3. Results and Discussion

### 3.1. Amylose Content and Hydration Properties

The amylose content and hydration properties (WAC, WAI, WSI, and SP) are presented in Table 2. Amylose concentrations ranged from 2.78 to 22.08%. As expected, waxy maize and waxy rice samples showed the lowest amylose concentrations, whereas wheat and normal maize showed the highest values, followed by tapioca, potato, and normal rice. Previous works have reported the following amylose concentrations in starches: maize ranging from 0% (waxy) to 40%, rice from 0% (waxy) to 30% [30], wheat from 13 to 32% [16,32], potato from 18 to 28% [33,34,35,36], and tapioca from 15 to 25% [37,38,39].

The WAC values allowed grouping the samples into two main groups: one containing wheat, waxy rice, normal rice, and tapioca that showed the lowest values (0.82–0.89 g/g), and one containing normal maize, waxy maize, and potato that showed the higher values (1.01–1.08 g/g). The variation in WAC could be due to variations in starch structure, resulting in variations in the formation of covalent and hydrogen bonds between the starch chains and, thereby, in the availability of water binding sites [23,40]. WAI allowed the samples to be divided into three groups: potato and waxy maize, which showed the lowest values (4.2–4.3 g/g), followed by normal maize, tapioca, and wheat (7.0–8.0 g/g), and finally normal rice and waxy rice, which showed the highest results (12.4–12.5 g/g). The thermal contribution of the test dissociated the hydrogen bridge interactions of the starch, allowing the hydroxyl groups of the polysaccharide to interact with water, thereby increasing the solubility and swelling of the granules [30]. Swelling power (SP), which followed the same trend as WAI, is linked to the ability of starch granules to absorb water during gelatinization [41]. Solubility (WSI) and swelling power reflect the interaction between the amorphous and crystalline regions of starch granules [30,42]. Waxy rice and normal rice showed the highest SP and WAI values, indicating that the difference in amylose concentration did not substantially modify the water binding capacity of these starch samples, a behavior that could be explained by the similar structure of amylopectin in both samples [30]. These two starches also had the same and the lowest WSI values, reinforcing the idea that the amount of amylose does not exert the main effect on the swelling and solubilization of the starch granules of these two samples. On the contrary, waxy and normal maize starches showed the most marked differences in WSI values, with the waxy type showing the highest solubility and the normal maize presenting one of the lowest values. Because all studied samples were of different sources and natures, it is not possible to use amylose content alone to predict swelling capacity and solubility, as these variables are also influenced by the structure of amylopectin and the extent of interaction between the amorphous and crystalline zones [30,43,44]. The extension and ability of starch granules to swell is mainly a property of their amylopectin content, but amylose may also have an inhibiting or delaying effect on it [45]. 

### 3.2. Thermal Properties

The results from the DSC analyses are presented in Table 3. Potato starch showed the highest enthalpy of gelatinization (18.0 J/g), followed by the waxy starch samples (16.2 J/g (rice) and 15.68 J/g (maize)). Wheat starch had the lowest gelatinization enthalpy, following the general premise that samples with higher amylose content usually have lower ΔH(G) values [37]. Potato starch is reported to have long external chains on the amylopectin molecule, which exert a higher enthalpy of gelatinization in the granule [31]. The elevated ΔH(G) in potato starch might also be explained by its high crystallinity; thus, more energy is required to break it down [46].

Wheat starch showed the lowest onset temperature of gelatinization (To), followed by potato and waxy rice, whereas normal rice had the highest value. In rice starch samples, a higher amylose content has been correlated with a higher gelatinization temperature [47]; however, this correlation was not found in other starch samples, such as wheat starch or normal and waxy maize, suggesting that the internal structure of the starch granule exerts an important effect on the gelatinization process [14,48].

In general, peak (Tp) and ending (Te) temperatures of gelatinization varied among starches in the same order as To, with some exceptions due to the different shapes and widths (ΔT = Te − To) of the gelatinization peaks. Potato recorded the narrowest gelatinization peak (ΔT = 9.2 °C), whereas waxy rice showed the widest (19 °C), denoting a more complex and heterogeneous crystalline structure.

The enthalpy of the melting of amylopectin recrystallized after 7 days of storage at 2 ± 2 °C; the DR and the temperatures of this transition are also shown in Table 3. The highest DR values were observed for potato, normal maize, and wheat starches, highlighting that the non-waxy cereal samples (wheat, normal maize, and normal rice) did not show statistical differences between their DR values. The double helices formed by amylopectin are shorter than those formed by amylose due to the branched structure and branch lengths of amylopectin [30]; thus, amylopectin molecules with longer chains, as in the case of potato starch, would form more stable structures and would be expected to have a greater value of retrogradation enthalpy. The values of DR are related to the ease of rearrangement between starch molecules in which samples with higher amounts of amylose, such as non-waxy cereal samples, have a higher retrogradation capacity [49], and they would be expected to show higher percentages of this parameter. The waxy rice samples showed the lowest DR values, which can be attributed to a higher proportion of A-chains and shorter external chains in the amylopectin molecule [50]. The retrogradation peaks showed lower To values and higher ΔT values than those corresponding to the gelatinization scans, which is similar to other samples of starchy products tested previously [29,51,52,53], suggesting that fewer perfect crystalline regions were formed during storage [54,55]. The highest ΔT values of retrogradation were observed in potato (38 °C) and waxy rice (36 °C) starches, whereas the other samples ranged between 25 and 29 °C. 

The amylose–lipid complex dissociation peak, which appeared in the range of 85 and 105 °C, was only recorded in normal maize, normal rice, and wheat starches. Normal rice showed the highest amylose–lipid dissociation enthalpy. As expected, it was not detected in waxy starches due to their low amylose content; it was not observed in tuber starches such as potato and tapioca either, despite their significant amounts of amylose. The occurrence of amylose–lipid complex dissociation peaks is common in cereals, which may contain about 1% lipid content, whereas starches from tubers, legumes, and waxy starches contain practically no lipids [56]; thus, values for samples such as potato are very low or undetectable [48]. 

### 3.3. Pasting Profiles

**Figure 1:**
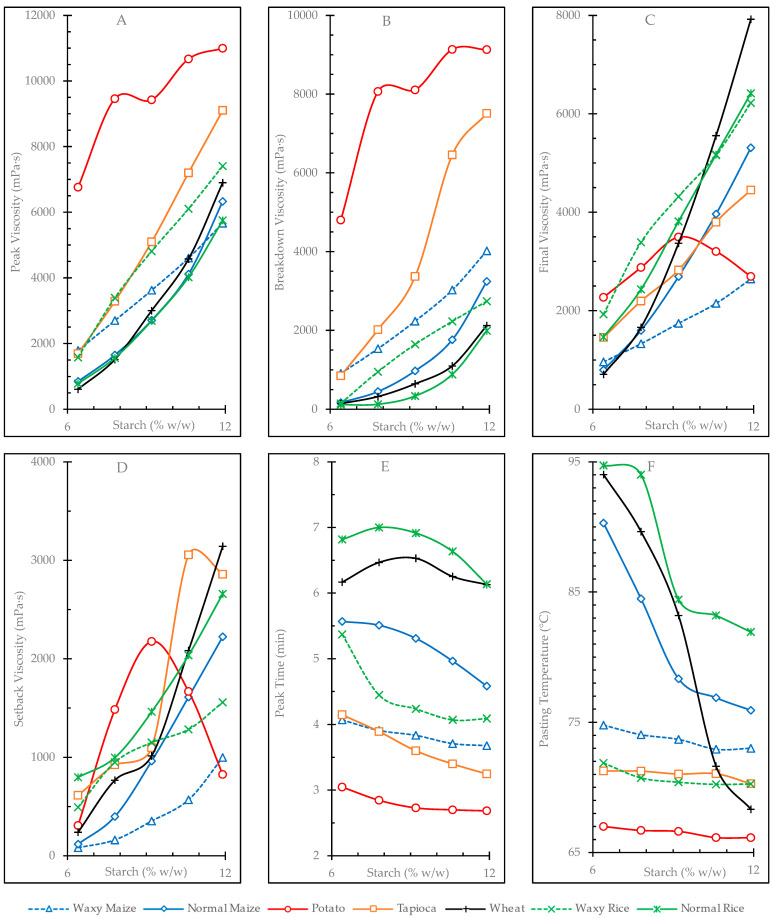
Effect of starch concentration on pasting parameters. (**A**) = Peak viscosity (PV); (**B**) = breakdown viscosity (BV); (**C**) = final viscosity (FV); (**D**) = setback viscosity (SV); (**E**) = peak time (Pt); (**F**) = pasting temperature (PT).

The pasting properties of the starches at all concentrations studied are presented in Table S1. The evolution of pasting parameters with concentrations are presented in Figure 1. The values obtained from fitting this evolution to a potential equation, P = k∙C^e^, are summarized in Table 4. The dependence of pasting viscosities (PV, TV, BV, FV, and SV) on starch concentration, in general, fit well to the potential model (with R^2^ coefficients ranging 94–99) except for potato starch, which was always far from this potential evolution, and tapioca starch, which did not follow it in the cases of TV and SV. This procedure facilitates the comparison between starches regarding the effect that concentration has on their pasting properties by using only two values: the fitting coefficient *k* (which quantifies the value of the evaluated property, P, at a concentration of 1 g/100 g) and the exponent *e* (which informs the rate of increase of the P parameter with concentration).

Potato starch gels showed the highest PV values (6763–10,998 mPa·s), which presented significantly equal values at concentrations ≥9.2 g/100 g. This can also be evaluated from its very high *k* coefficient for PV and the very low *e* exponent, indicating little dependence of this variable on concentration. Normal rice, normal maize, and wheat starches showed the lowest PV values at low concentrations and the highest rate of increase with concentration, as evidenced by their low *k* coefficient values and high *e* exponents (Table 4). On the contrary, potato, waxy maize, and waxy rice starches had the highest PV values at low concentration and the lowest rates of increase with concentration. When starch undergoes gelatinization and its granules become larger because of swelling, amylose is released and forms a network around the swollen structures, restricting the final swelling of the granules and peak viscosity [14]. In this sense, when comparing starches of the same botanical origin, such as natural maize or rice and their waxy counterparts, higher amylose content usually leads to a decrease in PV [57,58,59,60]. However, pasting properties are affected by many factors in addition to amylose content, such as lipid and phosphorus content, starch granule size, the branch chain length distribution of amylopectin, and the molecular size of amylose [60]. It was observed that the volume of gluten-free breads correlated positively with the peak viscosity of the flour [61]; thus, this parameter could be used to predict the quality of the product made with these ingredients. The amount of raw material should also be considered for the preparation of gluten-free products because some materials register more pronounced changes in PV than others as the concentration changes.

Potato starch also showed the highest breakdown viscosity (BV) values (4801–9129 mPa·s) denoting its lowest stability against heating and stirring (Figure 1B). Breakdown in the non-waxy cereal and tapioca samples showed a greater dependence on concentration, as indicated by their higher exponent *e* (Table 4). A significant negative correlation has been reported between breakdown viscosity and the specific volume of gluten-free breads [62]. It has been reported that waxy starches provide textures with good acceptability to the processed products [16,17], but they also show high breakdown values; thus, the amounts of them used in the formulations must be balanced to obtain food products of higher quality. The concentration of starches in the formulations could also be important, as the BV values in some samples showed important changes when changing concentration.

FV increased potentially with concentration in all studied starches (see Table 4 and R^2^ values >98%) except potato starch. As shown in Figure 1C, the FV of potato starch followed a parabolic evolution, presenting its maximum at a concentration of 9.2%. Within the other samples, the results are consistent with the general idea that starches with higher amylose content (such as wheat, normal maize, and normal rice) generally have higher FV values (usually measured at concentrations close to 10%) [32,63]. The highest FV at the gel concentration of 11.9 g/100 g corresponded to samples with higher amylose content (wheat (7922 mPa·s) and normal rice (6223 mPa·s)). 

The SV that results from the difference FV − TV is related to amylose retrogradation, and it is generally lower in waxy or low-amylose content starches [2,63,64,65]. In the studied samples, SV behaved differently according to the starch concentration in the gel (Figure 1D), which is similar to what was determined for FV. At higher concentrations of gels, their overall behavior was closer to predicted: samples with higher amylose content (wheat, normal rice and normal maize) showed higher SV values, whereas waxy samples had lower values. Tapioca and potato did not follow the expected SV trend with respect to amylose content, a behavior that is also shown in other parameters studied in this work, which could be explained by the presence of long amylopectin chains [66,67] and the high crystallinity of the samples [68], which reduce the effect of amylose on the mentioned parameters. 

The potato starch gels followed an inverted U-shaped trend in SV values with increasing concentration, showing a maximum value (2176 mPa-s) at 9.2% *w*/*w* (Figure 1D), which does not allow a proper fit to a potential equation (Table 4). This behavior indicates that potato starch could reach saturation conditions where parameters such as the starch–water interaction would be different, and the effect of concentration would not be equal to that observed at low concentrations. Tapioca starch had the highest SV with respect to the other starches at a concentration of 10.6% *w*/*w* (3056 mPa·s), a value that then decreased at 11.9% *w*/*w*, indicating that tapioca starch, like potato starch, experienced saturation behavior at high gel concentrations.

Increasing the concentration of the gels tends to maintain or decrease the peak time (Pt), with negative *e* exponents and values <1 (Table 4). Among all of the parameters measured during pasting events, Pt changed the least with changes in concentration. As a general trend, the samples with the lowest Pt values were those with the lowest amylose content; however, tuber starches did not follow this trend, showing the lowest peak times at all concentration levels (Figure 1E). Cereal samples with higher amylose content (normal rice, wheat, and normal maize) showed the highest Pt values at all concentration levels. 

Pt tended to decrease with increasing gel concentration in all cases (Figure 1F, Table 4). The non-waxy cereal samples (wheat, normal maize and normal rice) showed a strong dependence of Pt on gel concentration (given by the coefficient *e*) and on high Pt values at each concentration, which is explained by the high coefficient *k*. Samples with lower amylose content registered variations of Pt ~1 °C between the lowest and highest gel concentration values (1.65 °C and 1.86 °C for waxy rice and waxy maize, respectively). A similar behavior was observed for tuber starches (0.86 °C and 0.98 °C for potato and tapioca, respectively), showing once again the low dependence of the measured parameters on the amylose content of these two samples. On the other hand, non-waxy cereal samples with higher amylose content showed a marked decrease in Pt with increasing starch concentration in the gels. Thus, wheat starch recorded the highest difference in Pt (25.71 °C) between the 6.4 and 11.9% *w*/*w* concentration gels, followed by normal maize (14.39 °C) and normal rice (12.80 °C). This marked change in the cereal samples shows more clearly that as gel concentration increases, amylose loses the ability to delay granule swelling, requiring a lower temperature to initiate the pasting process. The PT provides important information about the quality of gluten-free bakery products. A significant positive correlation between the specific volume of bread and PT has been reported [62]. Given the results presented in this work, not only is PT relevant in the selection of starches for food processing, but also the concentration of the starch selected in the formulation, as some starches can have very important variations at different ratios during processing.

### 3.4. Rheological Properties of Gels

#### 3.4.1. Oscillatory Frequency Sweep Tests

**Figure 2:**
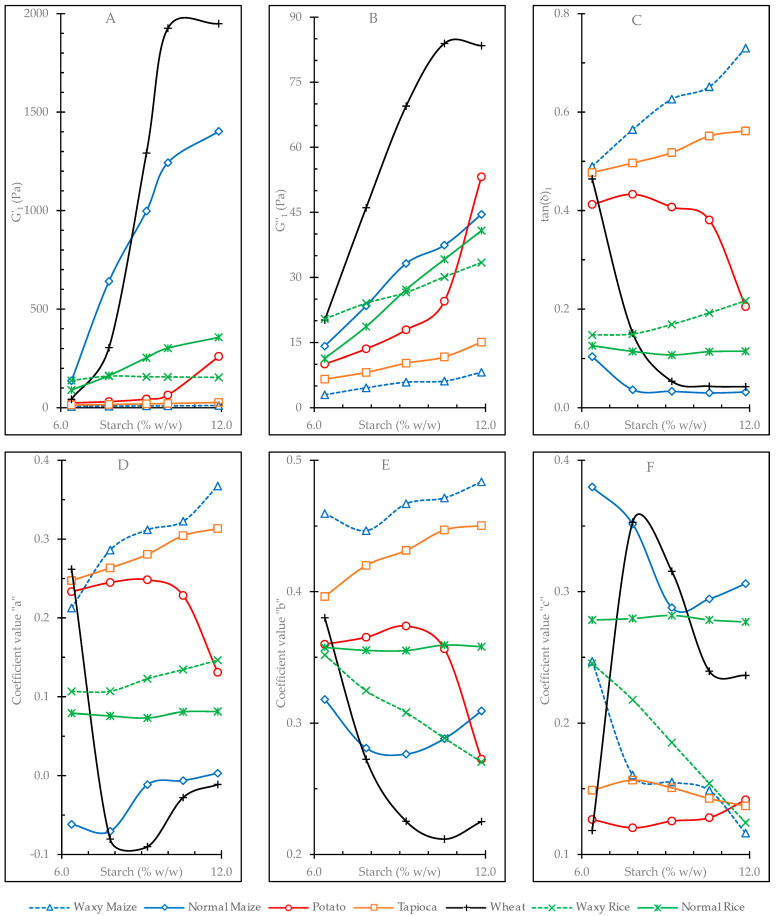
Effect of starch concentration on rheological properties obtained from frequency sweep tests. (**A**) = G′_1_; (**B**) = G″_1_; (**C**) = tan (δ)_1_; (**D**) = *a* exponent; (**E**) = *b* exponent; (**F**) = *c* exponent. All of these parameters were obtained by fitting the frequency sweep data to a power law model: G′ = G′_1_·F^a^; G″ = G″_1_·F^b^; tan (δ) = tan (δ)_1_·F^c^, where F is the oscillation frequency (Hz).

The evolution of the G′_1_, G″_1_, and tan δ_1_ coefficients and *a*, *b*, and *c* exponents (obtained from the frequency sweeps tests) versus gel concentration is illustrated in Figure 2 and presented in Table S2. Table 5 summarizes the parameters obtained from fitting this evolution to a potential equation. As shown in Table 5, some viscoelastic properties of some starches (the elastic modulus for waxy rice gels and the loss tangent for normal rice and potato gels) did not show an acceptable fit to the potential model (R^2^ < 84).

In all cases, the values determined for the elastic modulus (G′_1_) were higher than those of the viscous modulus (G″_1_), resulting in a loss tangent of (tan (δ)) < 1, indicating an elastic-like behavior in the studied gels. The value of G′_1_ in each material increased with concentration, where its evolution was influenced by the nature of each starch. 

Wheat starch gels showed the largest increases in G′_1_ and G″_1_ with increasing concentration (Figure 2A,B), which is reflected by the high *e* exponents obtained from the fits to the potential equation (Table 5). The normal maize starch gels also showed large changes in G′_1_ with increasing concentrations, a phenomenon also observed in the changes in G″_1_, although its viscous modulus was surpassed by waxy rice gels at low concentrations (6.4 and 7.8%) and by potato starch gel at 11.9% (Figure 2B). In both G′_1_ and G″_1_, the waxy maize gels presented the lowest values, which resulted in low *k* coefficients and *e* exponents (Table 5), indicating little variation with changes in gel concentration.

At all concentration levels, waxy maize starch gels showed the highest tan(δ)_1_ values, which increased with concentration (Table S2, Figure 2C). Tapioca starch gels also exhibited high loss tangent values with an increasing evolution with concentration, denoting the lower elasticity of these gels compared to those of the other starches. Next, in decreasing order, were potato starch gels, but in this material, the tan(δ)_1_ values decreased with increasing concentration, indicating a more elastic behavior in the more concentrated gels. The wheat starch gels showed a very pronounced decrease in the loss tangent values with increasing concentration, which is reflected by the high negative value of the *e* exponent compared to the other samples. The gels made from normal maize starch, followed by those made from normal and waxy rice starches, showed the lowest tan(δ)_1_ values, denoting their more elastic behavior. The normal and waxy rice samples had close values at low concentration, but though the tan(δ)_1_ of waxy rice gels increased at higher concentrations, with an *e* exponent similar to that of waxy maize, the normal rice gels tended to decrease with concentration similarly to the other two non-waxy cereal starches (wheat and normal maize).

The evolution of the *a*, *b*, and *c* exponents (which quantify the dependence of G′, G″, and tan(δ) with frequency) with gel concentration is illustrated in Figure 2D–F, respectively. In general, the two samples with the lowest G′_1_ and G″_1_ moduli, waxy maize and tapioca, showed the highest *a* and *b* exponents at almost all concentration levels, whereas wheat and normal maize starch gels, with the highest viscoelastic moduli, had the lowest values. This indicates that gels of softer consistency are, in general, more dependent on frequency than those of stronger structure. The evolution of the *a*, *b*, and *c* exponents with concentration did not follow a clear trend. At high concentrations, potato starch gels showed a significant drop in their frequency dependence (concomitant with the increase in their viscoelastic moduli), whereas for wheat starch gels, the drop of these exponents was determined at lower concentrations. The values of coefficient *c* did not follow a particular pattern in wheat and normal maize starch gels (Figure 2F). Gels of normal rice starch had high *c* values, but theirs were always lower than those of normal maize gels, with no appreciable variation with concentration. The waxy samples showed the most significant drops in the *c* exponent with concentration, whereas the tapioca and potato gels showed low values, with little change at all studied concentrations. The *c* exponent represents the difference of the *b* and *a* exponents (*b* − *a*); thus, a low value of *c* indicates a similar dependence of both viscoelastic moduli on frequency.

#### 3.4.2. Oscillatory Deformation Sweep Tests

**Figure 3:**
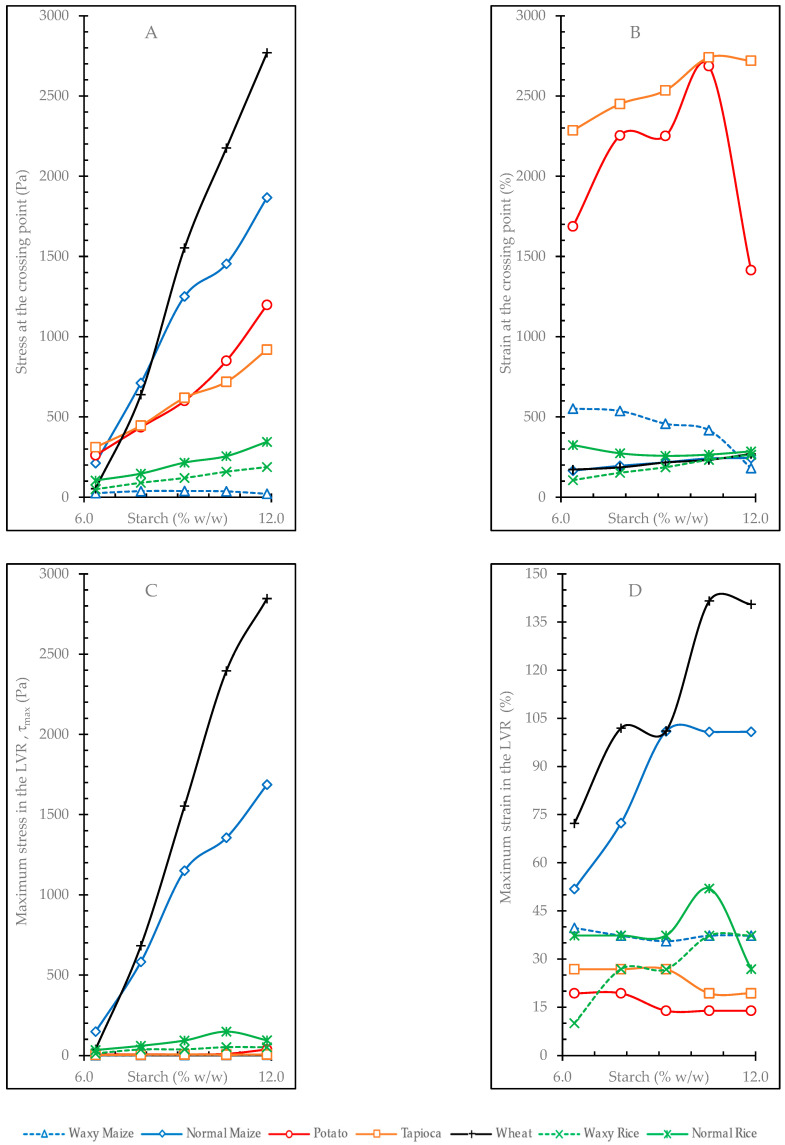
Effect of starch concentration on rheological parameters obtained from strain sweep tests. (**A**) = Stress at the crossing point (G′ = G″); (**B**) = strain at the crossing point; (**C**) = maximum stress (τ_max_); (**D**) = maximum strain.

The stress required to reach the crossover point (G′_1_ = G″_1_), where the structure of the gel passes from a predominantly elastic behavior to one with a more pronounced viscous character, and the corresponding deformation of the sample at this point in function of the gel concentration, are shown in Figure 3A,B, respectively. The parameters *k* and *e* obtained from fitting the evolution of these values with concentration to a potential equation are shown in Table 6.

The fits to the potential equation in the gels of wheat and normal maize starch were good in all cases, with R^2^ values greater than 80 (Table 6). The fits were poor for some parameters in Table 6, with R^2^ values located above 50 but below 80, as is the case of τ_max_ for normal rice and tapioca and of maximum strain in potato and tapioca. In the waxy maize gels, there was not an acceptable fit (R^2^ < 50) for crossing point stress and crossing point strain, which is possibly because of the low final viscosity values of the sample. Normal rice showed a lack of fit in both crossing point stress and maximum strain. Crossing point strain also showed non-adjustment in the potato gels, which is a consequence of the erratic behavior shown in Figure 3B.

With increasing concentration, the wheat gels showed a considerable increase in stress at the crossing point (Figure 3A), a behavior shared with the normal maize gels. This dependence of the stress value on concentration is reflected in the high exponents *e* of wheat (3.2) and normal maize (2.5). However, the strain reached at the crossover point by both wheat and normal maize gels did not show much variation with respect to concentration (Figure 3B); they had very low strain values and no significant differences between them (Table S3). This behavior indicates that with rising concentration, a significant increase in stress is necessary to reach similar values of deformation at the crossover point. Potato and tapioca gels also showed high *e* exponents in crossing point stress (2.5 and 1.7 respectively) but lower *k* coefficient values, which can be seen in Figure 3A, where the wheat and maize curves are followed by those of potato and tapioca. However, the potato and tapioca starch gels showed much higher deformations than the other samples (Figure 3B). The *k* coefficient of tapioca starch gels in crossing point strain was much higher than those of the other gels, although its low *e* exponent (Table 6) demonstrates a little dependence of the deformation value at the crossover point with concentration. The potato starch gels showed a significant drop of this deformation value at the maximum gel concentration (11.9%), which could be indicative of gel saturation, as reflected by the lack of fit in the potential equation.

The variations of the maximum stress that marks the end of the LVR and the corresponding maximum strain with the gel concentration are shown in Figure 3C,D, respectively. The fitting parameters of the evolution of these variables versus concentration to a potential equation are shown in Table 6. The wheat starch and normal maize starch gels showed the greatest change of maximum stresses with concentration, with *e* exponents of 3.2 and 2.6, respectively. These starches also showed the highest maximum strain values, displaying high *e* exponents and *k* coefficients. These data indicate that increasing the concentration in the wheat and normal maize starch gels would allow for higher maximum stress (τ_max_) and maximum strain before their structures are destroyed. 

### 3.5. Principal Components Analysis

**Figure 4:**
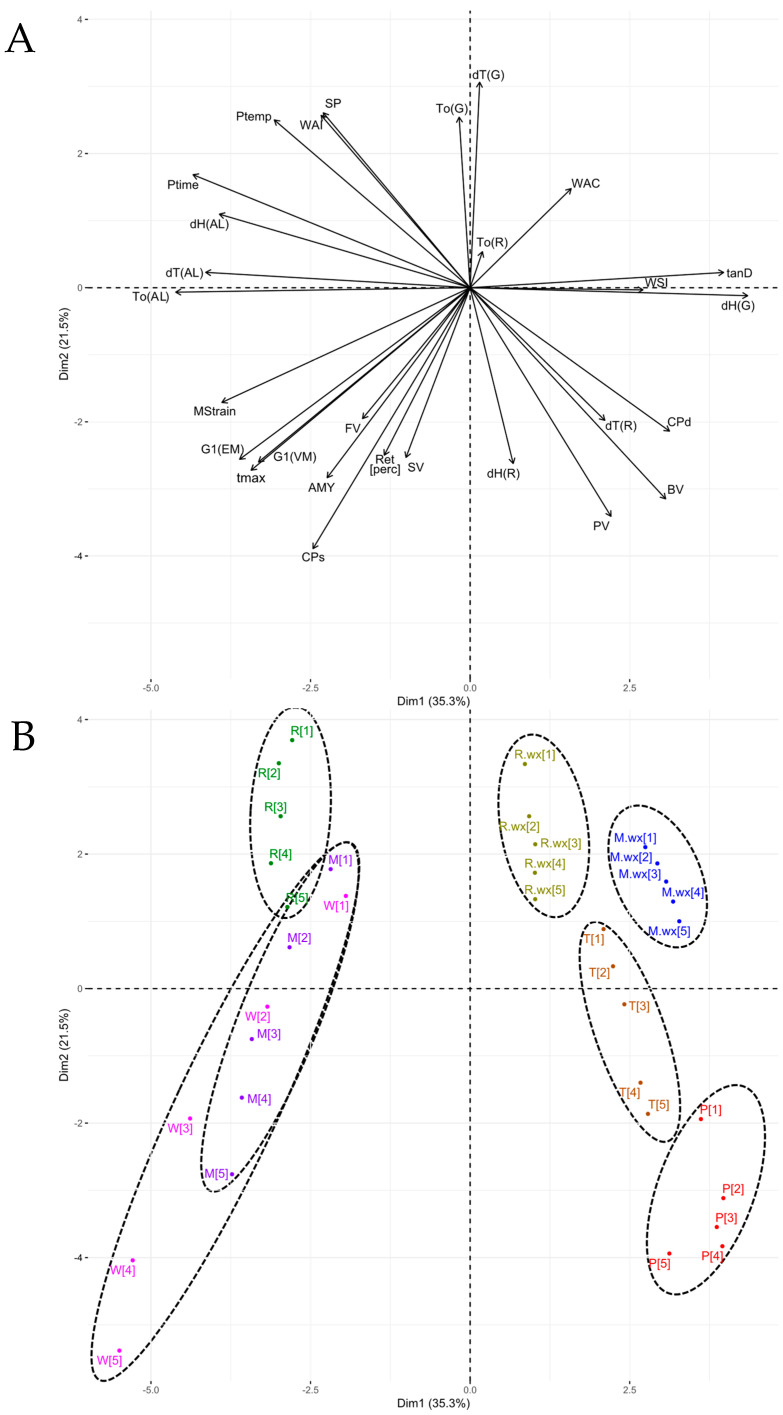
Principal Components Analysis. (**A**) Variables: AMY = amylose content; BV = breakdown viscosity; CPd = crossing point deformation; CPs = crossing point stress; dH(AL) = enthalpy of dissociation of the amylose–lipid complex; dH(G) = enthalpy of gelatinization; dH(R) = enthalpy of retrogradation; dT(AL) = ΔT of amylose-lipid complex; dT(G) = ΔT of the gelatinization peak; dT(R) = ΔT of retrogradation peak; FV = final viscosity; G1(EM) = G′_1_; G1(VM) = G″_1_; MStrain = maximum strain; Ptemp = PT; Ptime = Pt; PV = peak viscosity; Ret[perc] = % of retrogradation (DR); SP = swelling power; SV = setback viscosity; tanD = tan(δ)_1_; tmax = maximum stress within the LVR (τ_max_); To(AL) = onset temperature of the amylose–lipid complex; To(G) = onset temperature of the gelatinization peak; To(R) = onset temperature of the retrogradation peak; WAC = water absorption capacity; WAI = water absorption index; WSI = water solubility index. (**B**) Samples: M = normal maize; M.wx = waxy maize; P = potato; R = normal rice; R.wx = waxy rice; T = tapioca; W = wheat. Bracketed numbers from [1] to [5] represent the five gel concentration levels in ascending order.

The conducted principal component analysis (PCA) is illustrated in Figure 4, with starch samples identified with different colors and variables represented in black.

The model explains 56.8% of the total variation between samples. It consists of two dimensions, where Dim1 is presented in the horizontal axis and Dim2 in the vertical axis. The central point of the graph, which defines four quadrants, represents the zero-contribution value from which the studied parameters emerge as vectors, depending on whether they are positively or negatively correlated with each of the two dimensions.

Cereal samples with higher amounts of amylose (wheat, normal rice, and normal maize) are located in the zone of negative Dim1 values, where the greatest effect of amylose concentration and calorimetric parameters of dissociation of the amylose–lipid complex are found. The wheat starch gels at different concentrations show an enormous dispersion along Dim1, which is related to the pronounced separation of pasting temperature with increasing concentration as well as a stronger relationship of the more concentrated gels with the vectors of FV, SV and some rheological parameters (maximum stress, maximum strain, G′_1_, G″_1_ and crossing point stress), which is in agreement with the high *e* coefficients shown by this starch in these parameters. The normal maize starch gels have a very similar distribution on the graph as wheat starch, but they are less dispersed, whereas normal rice starch has a much narrower distribution. With respect to the range of positive Dim1 values, waxy rice and waxy maize samples were placed in the positive quadrant of Dim2, whereas potato starch gels are in the negative quadrant of Dim2, and tapioca starch gels are in an intermediate position. Gels of waxy samples, potato, and tapioca were found to be opposite to the effect of amylose concentration and are also shown to be less affected by parameters such as pasting temperature, crossing point stress, and maximum stress, which did not vary greatly with increasing concentrations in these four materials.

Regarding Dim2, tapioca and potato samples showed the highest values of deformation at the crossing point as well as the highest values of PV. With respect to the latter variable, wheat starch samples showed the greatest increase in PV with concentration, as evidenced by the evolution of the higher concentration gels towards negative Dim2 values, as did the PV vector. Potato, wheat, and normal maize samples showed the highest values in the retrogradation degree and high values in retrogradation enthalpy. The waxy maize and normal maize gels are in the positive quadrant of Dim 2, agreeing with the records of the highest values in WAI, SP, and ΔT(G) together with normal maize and tapioca gels. 

The high tan(δ)_1_ values in the waxy rice and tapioca gels coincide with the position of these samples in the vicinity of the vector of this variable. Potato starch gels showed lower tan(δ)_1_ values for samples of higher concentration, which is illustrated by the greater distance of the higher concentration gels to the vector of this variable. The waxy starch gels, together with potato and tapioca, had the highest ΔH(G) values, justifying the closeness of these samples to the vector of this variable. 

## 4. Conclusions

The concentration of starch gels influences their viscoelastic behavior in different ways. The gels that underwent the greatest changes were those from non-waxy cereals, with wheat starch gels showing the greatest changes, followed by gels from normal maize. The amylose content influenced the behavior of maize gels when comparing the waxy and normal samples, whereas waxy and normal rice gels did not show such a great distance between the measured parameters, indicating that the molecular structure of the granule in rice samples have more weight in the explanation of the observed phenomena. Potato and tapioca gels showed different behaviors than cereal gels, being one of the most important differences in the little effect of amylose concentration on pasting properties, with similar results to waxy samples but with higher viscosities. The structure of the amylopectin chains probably also exerts an important effect on these parameters. The calorimetric parameters and the hydration properties allowed for a better clustering of the parameters in the PCA, helping to determine the characteristics of each starch and contributing to the inference of the behavior that each material would have in the elaboration of foods and, in particular, gluten-free products.

## Figures and Tables

**Table 1:** Relationship between weighed starch masses and their corresponding concentrations.

Starch Weight (1)	Paste Concentration (2)
2.0	6.4
2.5	7.8
3.0	9.2
3.5	10.6
4.0	11.9

(1) Expressed as g starch corrected to 14% moisture content in 25 g of water. (2) Expressed in g starch/100 g suspension.

**Table 2:** Amylose content and hydration properties of flour samples.

Sample	Amylose (%)	WAC ^(1)^	WAI ^(2)^	WSI ^(3)^	SP ^(4)^
Wheat	22.1 ± 0.3 ^a^	0.82 ± 0.01 ^b^	8.0 ± 0.4 ^b^	1.8 ± 0.5 ^c^	8.2 ± 0.4 ^b^
Normal maize	19.8 ± 0.8 ^a,b^	0.82 ± 0.03 ^b^	7.0 ± 0.1 ^c^	0.7 ± 0.1 ^d,e^	7.0 ± 0.1 ^c^
Waxy maize	2.8 ± 0.1 ^d^	1.07 ± 0.02 ^a^	4.2 ± 0.1 ^d^	7.1 ± 0.8 ^a^	4.4 ± 0.1 ^d^
Normal rice	15.0 ± 1.0 ^c^	1.08 ± 0.01 ^a^	12.5 ± 0.1 ^a^	0.12 ± 0.05 ^e^	12.5 ± 0.1 ^a^
Waxy rice	2.8 ± 0.2 ^d^	0.89 ± 0.01 ^b^	12.4 ± 0.1 ^a^	0.03 ± 0.02 ^e^	12.4 ± 0.1 ^a^
Potato	17.9 ± 0.1 ^b,c^	1.01 ± 0.06 ^a^	4.3 ± 0.1 ^d^	1.7 ± 0.2 ^c,d^	4.3 ± 0.1 ^d^
Tapioca	18.7 ± 0.2 ^b^	0.83 ± 0.01 ^b^	7.4 ± 0.4 ^b,c^	4.5 ± 0.4 ^b^	7.7 ± 0.4 ^b,c^

^(1)^ g water/g starch; ^(2)^ g gel/g starch; ^(3)^ g soluble solids/100 g starch; ^(4)^ g gel/g insoluble starch. The presented data are means ± standard deviations. Different letters in each column indicate significant differences between means at *p* < 0.05.

**Table 3:** Thermal properties of starches at the studied concentrations.

	Wheat	Normal Maize	Waxy Maize	Normal Rice	Waxy Rice	Potato	Tapioca
Gelatinization							
ΔH (J/g)	10.7 ± 0.1 ^e^	12.4 ± 0.1 ^d^	15.7 ± 0.1 ^b,c^	13.0 ± 0.5 ^d^	16.2 ± 0.8 ^b^	18.0 ± 0.1 ^a^	14.6 ± 0.1 ^c^
To (°C)	53.1 ± 0.1 ^e^	63.1 ± 0.1 ^b^	63.4 ± 0.1 ^b^	67.9 ± 0.1 ^a^	57.5 ± 0.2 ^d^	57.4 ± 0.1 ^d^	61.3 ± 0.1 ^c^
Tp (°C)	58.6 ± 0.1 ^f^	68.7 ± 0.1 ^b^	69.0 ± 0.1 ^b^	74.9 ± 0.1 ^a^	66.5 ± 0.1 ^c^	61.4 ± 0.1 ^e^	65.9 ± 0.1 ^d^
Te (°C)	63.5 ± 0.1 ^e^	75.3 ± 0.2 ^b^	76.4 ± 0.2 ^b^	80.4 ± 0.2 ^a^	77 ± 1 ^b^	66.6 ± 0.1 ^d^	72.5 ± 0.1 ^c^
ΔT (°C)	10.4 ± 0.1 ^b,c^	12.2 ± 0.2 ^b^	13.0 ± 0.2 ^b^	12.5 ± 0.3 ^b^	19 ± 1 ^a^	9.2 ± 0.1 ^c^	11.2 ± 0.1 ^b,c^
Retrogradation							
ΔH (J/g)	5.6 ± 0.9 ^b^	6.7 ± 0.3 ^b^	5.9 ± 0.1 ^b^	6 ± 1 ^b^	1.3 ± 0.5 ^c^	11.4 ± 0.1 ^a^	4 ± 1 ^b,c^
To (°C)	35 ± 2 ^a^	35.2 ± 0.4 ^a^	37.9 ± 0.3 ^a^	37 ± 1 ^a^	27.0 ± 0.7 ^b^	34 ± 2 ^a^	36.7 ± 0.1 ^a^
Tp (°C)	49.4 ± 0.1 ^b^	50.2 ± 0.2 ^b^	49.6 ± 0.7 ^b^	51.6 ± 0.9 ^b^	52 ± 4 ^b^	61 ± 1 ^a^	50 ± 2 ^b^
Te (°C)	63.8 ± 0.3 ^b^	63.9 ± 0.8 ^b^	63.2 ± 0.2 ^b^	63.5 ± 0.2 ^b^	63.3 ± 0.2 ^b^	72.8 ± 0.3 ^a^	64 ± 2 ^b^
ΔT (°C)	29.0 ± 0.9 ^b^	28.6 ± 0.3 ^b^	25.4 ± 0.5 ^b^	26 ± 1 ^b^	36.2 ± 0.9 ^a^	39 ± 2 ^a^	27 ± 2 ^b^
DR (%)	52 ± 7 ^a,b^	54 ± 2 ^a,b^	38 ± 1 ^c,d^	49 ± 7 ^b,c^	8 ± 3 ^e^	63 ± 1 ^a^	26 ± 7 ^d^
Amylose–lipid complex							
ΔH (J/g)	1.1 ± 0.1 ^b^	1.4 ± 0.1 ^b^	-	2.6 ± 0.1 ^a^	-	-	-
To (°C)	96.7 ± 0.4 ^a^	85 ± 1 ^c^	-	92.3 ± 0.1 ^b^	-	-	-
Tp (°C)	102.0 ± 0.2 ^a^	96 ± 1 ^b^	-	99.9 ± 0.3 ^a^	-	-	-
Te (°C)	104.7 ± 0.5 ^a^	102.5 ± 0.1 ^b^	-	104.6 ± 0.1 ^a^	-	-	-
ΔT (°C)	8.0 ± 0.1 ^c^	17 ± 1 ^a^	-	12.3 ± 0.2 ^b^	-	-	-

To = onset temperature; Tp = peak temperature; Te = ending temperature; ΔT = Te − To; ΔH = enthalpy of the phase transition (expressed in J/g of dry matter); DR = degree of retrogradation, calculated as the quotient between the enthalpy of retrogradation and the enthalpy of gelatinization. The presented data are means ± standard deviations. Different letters in each row indicate significant differences between means at *p* < 0.05.

**Table 4:** Coefficients from fitting the pasting parameters as a function of concentration to a potential equation. Formula: P = k∙C^e^, where P represents the different pasting parameters expressed in mPa·s (PV, TV, BV, FV, and SV), min (Pt), or °C (PT), and C represents the concentration of the gel expressed in g starch/100 g gel.

	Wheat	Normal Maize	Waxy Maize	Normal Rice	Waxy Rice	Potato	Tapioca
Peak Viscosity (PV)							
k	2.9 ± 2	1.9 ± 0.5	67 ± 6	2.9 ± 0.4	46 ± 24	2165 ± 784	21 ± 7
e	3.2 ± 0.2	3.3 ± 0.1	1.8 ± 0.1	3.1 ± 0.1	2.1 ± 0.2	0.7 ± 0.1	2.5 ± 0.1
R^2^	98.4	97.6	99.9	99.9	97.6	86.3	99.4
Trough Viscosity (TV)							
k	5 ± 3	10 ± 1	160 ± 42	12 ± 7	70 ± 21	2024 ± 1822	402 ± 727
e	2.9 ± 0.2	2.4 ± 0.01	1.0 ± 0.1	2.4 ± 0.2	1.7 ± 0.1	−0.2 ± 0.4	0.6 ± 0.7
R^2^	97.8	99.8	96.3	97.5	98.7	1.8	12.8
Breakdown Viscosity (BV)							
k	0.10 ± 0.01	0.10 ± 0.01	12.8 ± 1.3	0.10 ± 0.01	4 ± 4	1341 ± 836	4 ± 4
e	4.4 ± 0.4	4.9 ± 0.1	2.40 ± 0.01	6.9 ± 0.3	2.8 ± 0.4	0.8 ± 0.2	3.1 ± 0.4
R^2^	97.8	99.8	99.9	99.7	94.5	76.4	96.4
Final Viscosity (FV)							
k	3 ± 2	5 ± 1	47 ± 3	24 ± 7	108 ± 39	1533 ± 1172	58 ± 10
e	3.2 ± 0.2	2.9 ± 0.1	1.7 ± 0.1	2.3 ± 0.1	1.7 ± 0.1	0.3 ± 0.3	1.8 ± 0.1
R^2^	98.8	99.7	99.9	99.5	97.9	21.2	99.6
Setback Viscosity (SV)							
k	0.3 ± 0.1	0.3 ± 0.2	0.10 ± 0.01	13 ± 4	40 ± 22	346 ± 1030	4 ± 7
e	3.9 ± 0.2	3.7 ± 0.3	4.3 ± 0.1	2.2 ± 0.1	1.5 ± 0.2	0.6 ± 1.3	2.8 ± 0.8
R^2^	99.2	98.5	99.7	99.2	94.1	9.3	83.4
Peak Time (Pt)							
k	6.7 ± 0.9	10 ± 2	5.6 ± 0.1	9 ± 2	12 ± 3	4.5 ± 0.3	8.8 ± 0.2
e	−0.10 ± 0.01	−0.30 ± 0.01	−0.2 ± 0.01	−0.20 ± 0.01	−0.5 ± 0.1	−0.3 ± 0.01	−0.5 ± 0.01
R^2^	3.5	85.3	98.4	51.5	85.1	90.5	99.6
Pasting Temperature (PT)							
k	246 ± 36	156 ± 13	81 ± 1	158 ± 19	77 ± 2	70.0 ± 0.5	74 ± 1
e	−0.60 ± 0.01	−0.30 ± 0.01	−0.10 ± 0.01	−0.30 ± 0.01	−0.10 ± 0.01	−0.10 ± 0.01	−0.10 ± 0.01
R^2^	94.9	95.3	95.2	88.1	81.7	93.4	64

**Table 5:** Coefficient from fitting the frequency sweep parameters as a function of concentration to a potential equation. Formula: P = k∙C^e^, with P expressed in Pa (G′_1_ and G″_1_) or dimensionless (tan(δ)_1_).

	Wheat	Normal Maize	Waxy Maize	Normal Rice	Waxy Rice	Potato	Tapioca
G′_1_ (Elastic Modulus)							
k	1 ± 3	6 ± 7	1.4 ± 0.4	3 ± 2	118 ± 28	0.10 ± 0.01	1.9 ± 0.6
e	3.1 ± 0.9	2.3 ± 0.5	0.9 ± 0.1	2.0 ± 0.2	0.2 ± 0.1	9.5 ± 1.9	1.1 ± 0.1
R^2^	85.4	90.3	91.6	96.7	29.9	94.3	94.5
G″_1_ (Viscous Modulus)							
k	2 ± 1	0.8 ± 0.3	0.4 ± 0.1	0.4 ± 0.1	4.7 ± 0.2	0.10 ± 0.01	0.6 ± 0.1
e	1.7 ± 0.4	1.7 ± 0.1	1.3 ± 0.2	2.0 ± 0.1	0.80 ± 0.01	3.7 ± 0.7	1.4 ± 0.1
R^2^	86.3	97.2	90.9	98.9	99.8	91.1	97.5
tan(δ)_1_							
k	10^3^ ± 9^3^	17 ± 27	0.20 ± 0.01	0.20 ± 0.01	0.10 ± 0.01	2 ± 1	0.30 ± 0.01
e	−5.4 ± 0.5	−2.8 ± 0.8	0.6 ± 0.1	−0.2 ± 0.1	0.7 ± 0.1	−0.7 ± 0.4	0.30 ± 0.01
R^2^	99.2	83.5	91.7	38.0	92.9	49.0	97.7

**Table 6:** Coefficient of adjustment of deformation sweep parameters as a function of concentration to a potential equation. Formula: P = k∙C^e^, with P expressed in Pa (stress at the crossing point and max stress, τ_max_, within the LVR) or % (deformation at the crossing point and max strain within the LVR).

	Wheat	Normal Maize	Waxy Maize	Normal Rice	Waxy Rice	Potato	Tapioca
Crossing Point Stress							
k	1 ± 1	5.0 ± 5.1	25 ± 43	2.9 ± 0.8	1.7 ± 0.5	3.0 ± 0.8	14 ± 3
e	3.2 ± 0.5	2.5 ± 0.4	0.2 ± 0.7	2.0 ± 0.1	2.0 ± 0.1	2.5 ± 0.1	1.7 ± 0.1
R^2^	94.8	94.1	1.2	99.0	98.7	99.5	99.1
Crossing Point Deformation							
k	41 ± 8	53 ± 8	3^3^ ± 4^3^	481 ± 179	8 ± 1	2^3^ ± 3^3^	1.3^3^ ± 0.1^3^
e	0.80 ± 0.01	0.70 ± 0.01	−0.8 ± 0.6	−0.3 ± 0.1	1.50 ± 0.01	0.1 ± 0.6	0.30 ± 0.01
R^2^	96.8	96.6	35.7	39.3	99.5	0.3	94.6
Max Stress (τ_max_)							
k	1 ± 2	4 ± 4	0.3 ± 0.1	3 ± 6	1 ± 1	0.10 ± 0.01	1.8 ± 0.9
e	3.2 ± 0.6	2.6 ± 0.5	1.3 ± 0.2	1.6 ± 0.8	1.6 ± 0.4	9 ± 2	0.6 ± 0.2
R^2^	93.9	92.8	93.6	57.6	82.5	89.1	59.4
Max Strain							
k	11 ± 6	11 ± 7	48 ± 3	43 ± 52	1 ± 1	67 ± 28	79 ± 40
e	1.1 ± 0.2	1.0 ± 0.2	−0.20 ± 0.01	−0.1 ± 0.5	1.5 ± 0.4	−0.7 ± 0.1	−0.6 ± 0.2
R^2^	89.4	80.4	81.2	0.3	82.8	78.7	66.6

## Data Availability

All related data and methods are presented in this paper. Additional inquiries should be addressed to the corresponding author.

## Supplementary Materials

The following supporting information can be downloaded at: https://www.mdpi.com/article/10.3390/foods12122281/s1, Table S1: Pasting properties of starches at the different studied concentrations; Table S2: Rheological properties obtained from frequency sweeps of gels made with starches at different concentrations; Table S3: Deformation sweep profile at 1 Hz.
