# Supplementary material for: Impact of Starch Concentration on the Pasting and Rheological Properties of Gluten-Free Gels. Effects of Amylose Content and Thermal and Hydration Properties
Source: Foods. 2023 Jun 6;12(12):2281. doi: 10.3390/foods12122281 (PMC10297029; doi:10.3390/foods12122281)
Supplement: Supplementary file 1 [file foods-12-02281-s001.zip › foods-2392404-supplementary.pdf]

Table S1. Pasting properties of starches at the different studied concentrations.

Conc. (%)	PV (mPa·s)	TV (mPa·s)	BV (mPa·s)	FV (mPa·s)	SV (mPa·s)	Pt (min)	PT (°C)
Wheat							
6.4	613 ± 20^{eD}	470 ± 18^{eD}	143 ± 7^{cC}	709 ± 22^{fD}	240 ± 24^{eD}	6.1 ± 0.1^{bA}	94.0 ± 0.8^{aA}
7.8	2261 ± 812^{cdC}	1773 ± 638^{abC}	488 ± 176^{eC}	2515 ± 937^{abcC}	742 ± 299^{bcCD}	6.50 ± 0.05^{bA}	86 ± 3^{bB}
9.2	3002 ± 16^{dC}	2355 ± 40^{bC}	647 ± 34^{efBC}	3370 ± 33^{bcC}	1015 ± 13^{cC}	6.53 ± 0.01^{bA}	83.20 ± 0.05^{bB}
10.6	4576 ± 80^{dB}	3474 ± 132^{bB}	1102 ± 136^{fB}	5557 ± 115^{aB}	2083 ± 74^{bB}	6.2 ± 0.1^{bA}	71.6 ± 0.4^{dC}
11.9	6900 ± 52^{cdA}	4780 ± 38^{aA}	2120 ± 25^{eA}	7922 ± 66^{aA}	3142 ± 29^{aA}	6.13 ± 0.1^{aA}	68.3 ± 0.5^{eC}
Normal Maize							
6.4	850 ± 18^{dD}	680 ± 10^{dE}	170 ± 9^{cC}	799 ± 26^{eE}	119 ± 17^{fE}	5.57 ± 0.07^{cA}	90.3 ± 0.4^{bA}
7.8	1651 ± 17^{cdCD}	1201 ± 32^{bD}	450 ± 30^{eC}	1600 ± 23^{cdD}	399 ± 24^{cdD}	5.51 ± 0.03^{cA}	84.5 ± 0.4^{bB}
9.2	2709 ± 5^{dC}	1731 ± 23^{cC}	977 ± 18^{eBC}	2694 ± 25^{dC}	962 ± 6^{cC}	5.31 ± 0.03^{cA}	78.33 ± 0.03^{cC}
10.6	4115 ± 41^{eB}	2353 ± 91^{dB}	1762 ± 64^{eB}	3964 ± 71^{cB}	1611 ± 22^{cB}	4.97 ± 0.04^{cB}	76.9 ± 0.4^{bC}
11.9	6332 ± 110^{deA}	3087 ± 142^{bA}	3245 ± 66^{cdA}	5310 ± 149^{cA}	2223 ± 15^{bA}	4.58 ± 0.04^{bC}	75.9 ± 0.7^{bC}
Waxy maize							
6.4	1791 ± 19^{bE}	879 ± 9^{cB}	911 ± 10^{bE}	962 ± 10^{dE}	82 ± 5^{fB}	4.07 ± 0.01^{dA}	74.8 ± 0.5^{cA}
7.8	2700 ± 10^{bcD}	1165 ± 21^{bAB}	1535 ± 12^{cD}	1327 ± 1^{dD}	161 ± 21^{dB}	3.91 ± 0.03^{eB}	74.1 ± 0.5^{cAB}
9.2	3627 ± 45^{cC}	1393 ± 39^{dA}	2234 ± 40^{cC}	1741 ± 15^{eC}	348 ± 31^{dB}	3.84 ± 0.04^{eB}	73.7 ± 0.4^{dBC}
10.6	4605 ± 13^{dB}	1579 ± 54^{eA}	3026 ± 54^{cB}	2148 ± 62^{eB}	569 ± 115^{eAB}	3.71 ± 0.03^{eC}	72.9 ± 0.5^{cC}
11.9	5664 ± 51^{fA}	1645 ± 439^{cA}	4020 ± 411^{cA}	2643 ± 66^{eA}	998 ± 461^{cdA}	3.68 ± 0.05^{dC}	73.0 ± 0.5^{cC}
Normal rice							
6.4	784 ± 6^{dE}	666 ± 19^{dE}	118 ± 16^{cB}	1464 ± 26^{cE}	797 ± 17^{aD}	6.82 ± 0.07^{aA}	94.73 ± 0.05^{aA}
7.8	1565 ± 21^{dD}	1438 ± 11^{bD}	127 ± 19^{eB}	2435 ± 28^{abcdD}	997 ± 28^{bD}	7.0 ± 0.1^{aA}	94.0 ± 0.5^{aA}
9.2	2690 ± 22^{dC}	2352 ± 47^{bC}	338 ± 50^{fB}	3814 ± 33^{abC}	1462 ± 42^{bC}	6.92 ± 0.08^{aA}	84.4 ± 0.5^{aB}
10.6	4023 ± 120^{eB}	3137 ± 120^{cB}	886 ± 0^{fB}	5172 ± 135^{bB}	2035 ± 15^{bB}	6.64 ± 0.05^{aAB}	83.20 ± 0.07^{aB}
11.9	5752 ± 71^{efA}	3759 ± 78^{bA}	1993 ± 6^{eA}	6418 ± 200^{bA}	2659 ± 122^{abA}	6.14 ± 0.09^{aB}	81.9 ± 0.6^{aB}
Waxy rice							
6.4	1583 ± 43^{cE}	1435 ± 36^{bE}	148 ± 23^{cE}	1928 ± 34^{bE}	493 ± 21^{cD}	5.37 ± 0.26^{cA}	71.88 ± 0.05^{dA}
7.8	3392 ± 42^{bD}	2439 ± 58^{aD}	954 ± 16^{dD}	3389 ± 35^{aD}	950 ± 24^{bC}	4.45 ± 0.04^{dB}	70.7 ± 0.5^{cdB}
9.2	4812 ± 48^{bC}	3167 ± 62^{aC}	1645 ± 28^{dC}	4318 ± 37^{aC}	1151 ± 28^{bcB}	4.24 ± 0.04^{dB}	70.4 ± 0.4^{eB}
10.6	6106 ± 73^{cB}	3876 ± 53^{aB}	2230 ± 29^{dB}	5159 ± 70^{bB}	1284 ± 23^{dB}	4.07 ± 0.1^{dB}	70.23 ± 0.06^{eB}
11.9	7408 ± 11^{cA}	4666 ± 48^{aA}	2742 ± 59^{deA}	6223 ± 22^{bA}	1557 ± 49^{cA}	4.09 ± 0.03^{cB}	70.27 ± 0.03^{dB}
Potato							
6.4	6763 ± 160^{aC}	1962 ± 37^{aA}	4801 ± 131^{aB}	2270 ± 37^{aB}	308 ± 1^{dB}	3.05 ± 0.04^{eA}	67.00 ± 0.05^{eA}
7.8	9459 ± 395^{aB}	1394 ± 157^{bA}	8065 ± 544^{aA}	2877 ± 67^{abAB}	1483 ± 132^{aAB}	2.85 ± 0.04^{fB}	66.7 ± 0.6^{dAB}
9.2	9425 ± 286^{aAB}	1319 ± 225^{dA}	8106 ± 289^{aA}	3495 ± 497^{bA}	2176 ± 367^{aA}	2.73 ± 0.01^{gBC}	66.6 ± 0.5^{fAB}
10.6	10669± 215^{aAB}	1536 ± 235^{eA}	9133 ± 450^{aA}	3203 ± 266^{dAB}	1667 ± 31^{cAB}	2.70 ± 0.04^{gBC}	66.15 ± 0.1^{fAB}
11.9	10998± 751^{aA}	1869 ± 125^{cA}	9129 ± 793^{aA}	2695 ± 193^{eAB}	826 ± 219^{dAB}	2.69 ± 0.03^{fC}	66.14 ± 0.09^{fB}
Tapioca							
6.4	1693 ± 52^{bcE}	841 ± 27^{cB}	853 ± 38^{bD}	1454 ± 24^{cE}	614 ± 9^{bB}	4.15 ± 0.04^{dA}	71.3 ± 0.5^{dA}
7.8	3287 ± 28^{bD}	1270 ± 10^{bAB}	2017 ± 19^{bC}	2194 ± 19^{bcdD}	924 ± 11^{bB}	3.89 ± 0.03^{eB}	71.3 ± 0.3^{cA}
9.2	5096 ± 31^{bC}	1727 ± 31^{cA}	3369 ± 4^{bB}	2825 ± 73^{cdC}	1098 ± 103^{bcB}	3.60 ± 0.01^{fC}	71.03 ± 0.03^{eA}
10.6	7200 ± 54^{bB}	743 ± 23^{fB}	6457 ± 76^{bA}	3799 ± 37^{cB}	3056 ± 15^{aA}	3.40 ± 0.01^{fD}	71.05 ± 0.1^{deA}
11.9	9104 ± 9^{bA}	1593 ± 54^{cAB}	7511 ± 62^{bA}	4450 ± 83^{dA}	2857 ± 78^{abA}	3.25 ± 0.04^{eE}	70.3 ± 0.9^{dA}

Conc. (%) = Concentration, expressed as % w/w gel; PV = Peak Viscosity; TV = Trough Viscosity; BDV= Breakdown Viscosity; FV = Final Viscosity; SBV= Setback Viscosity; PT = Peak Time; PTemp = Pasting Temperature. The presented data are the mean ± standard deviation. Different letters in each column indicate significant differences between means at $p < 0.05$. Lowercase letters compare samples at the same concentration, while capital letters compare samples of the same botanical origin.

Table S2. Rheological properties obtained from frequency sweeps of gels made with starches at different concentrations.

**Conc. (%)**	**G'_1 (Pa)**	**a**	**G''_1 (Pa)**	**b**	**$\tan(\delta)_1$**	**c**
Wheat						
6.4	43 ± 1^{cB}	0.26 ± 0.01^{aA}	20 ± 1^{aC}	0.38 ± 0.01^{bcA}	0.46± 0.01^{bA}	0.12 ± 0.01^{cB}
7.8	304 ± 10^{bB}	-0.08 ± 0.01^{eB}	46 ± 2^{aB}	0.27 ± 0.01^{fB}	0.15± 0.01^{dB}	0.35 ± 0.01^{aA}
9.2	1292 ± 53^{aA}	-0.09 ± 0.01^{fB}	70 ± 2^{aA}	0.23 ± 0.01^{eB}	0.05± 0.01^{fB}	0.32 ± 0.01^{aAB}
10.6	1926 ± 149^{aA}	-0.03 ± 0.01^{eB}	84 ± 5^{aA}	0.21 ± 0.01^{dB}	0.04± 0.01^{fB}	0.24 ± 0.01^{bAB}
11.9	1949 ± 66^{aA}	-0.011 ± 0.001^{eAB}	83 ± 3^{aA}	0.23 ± 0.01^{fB}	0.04± 0.01^{eB}	0.24 ± 0.01^{bAB}
Normal Maize						
6.4	137 ± 6^{aD}	-0.06 ± 0.01^{dB}	14 ± 1^{bD}	0.32 ± 0.01^{eA}	0.10± 0.01^{fA}	0.38 ± 0.01^{aA}
7.8	641 ± 8^{aC}	-0.07 ± 0.01^{eB}	23 ± 1^{bC}	0.28 ± 0.01^{fA}	0.04± 0.01^{fB}	0.35 ± 0.01^{aAB}
9.2	998 ± 70^{bB}	-0.011 ± 0.001^{eA}	33 ± 2^{bB}	0.28 ± 0.01^{dA}	0.03± 0.01^{fB}	0.29 ± 0.01^{bC}
10.6	1244 ± 19^{bAB}	-0.006 ± 0.001^{eA}	37 ± 1^{bB}	0.29 ± 0.01^{cA}	0.03± 0.01^{fB}	0.29 ± 0.01^{aBC}
11.9	1403 ± 73^{bA}	0.003 ± 0.001^{eA}	45 ± 2^{cA}	0.31 ± 0.01^{dA}	0.03± 0.01^{eB}	0.31 ± 0.01^{aBC}
Waxy maize						
6.4	6 ± 1^{eC0}	0.21 ± 0.02^{bC}	3 ± 1^{eC}	0.46 ± 0.01^{aB}	0.49± 0.01^{aC}	0.25 ± 0.03^{bA}
7.8	8 ± 1^{eB}	0.29 ± 0.02^{aB}	5 ± 1^{fB}	0.45 ± 0.01^{aB}	0.57± 0.01^{aB}	0.16 ± 0.03^{dB}
9.2	9 ± 1^{eB}	0.31 ± 0.01^{aB}	6 ± 1^{fB}	0.47 ± 0.01^{aAB}	0.62± 0.015^{aB}	0.16 ± 0.01^{dB}
10.6	9 ± 1^{dB}	0.31 ± 0.01^{aB}	6 ± 1^{dB}	0.47 ± 0.01^{aAB}	0.62± 0.02^{aB}	0.16 ± 0.01^{cB}
11.9	11 ± 1^{eA}	0.37 ± 0.02^{aA}	8 ± 1^{fA}	0.48 ± 0.01^{aA}	0.73± 0.014^{aA}	0.12 ± 0.02^{cB}
Normal rice						
6.4	90 ± 9^{bD}	0.08 ± 0.01^{cA}	11 ± 1^{cE}	0.36 ± 0.01^{cdA}	0.13± 0.01^{eA}	0.28 ± 0.01^{bA}
7.8	164 ± 3^{cC}	0.08 ± 0.01^{dA}	19 ± 1^{cD}	0.36 ± 0.01^{dA}	0.11± 0.01^{eA}	0.28 ± 0.01^{bA}
9.2	254 ± 7^{cB}	0.07 ± 0.01^{dA}	27 ± 1^{cC}	0.36 ± 0.01^{bcA}	0.11± 0.01^{eA}	0.28 ± 0.01^{bA}
10.6	302 ± 22^{cB}	0.08 ± 0.01^{dA}	34 ± 1^{bcB}	0.36 ± 0.01^{bA}	0.11± 0.01^{eA}	0.28 ± 0.01^{aA}
11.9	357 ± 29^{cA}	0.08 ± 0.01^{dA}	41 ± 2^{cA}	0.36 ± 0.01^{cA}	0.12± 0.01^{dA}	0.28 ± 0.01^{aA}
Waxy rice						
6.4	139 ± 8^{aB}	0.11 ± 0.01^{cC}	20 ± 1^{aD}	0.35 ± 0.01^{dA}	0.15± 0.01^{dD}	0.25 ± 0.01^{bA}
7.8	161 ± 3^{cA}	0.11 ± 0.01^{cC}	24 ± 1^{bC}	0.33 ± 0.01^{eB}	0.15± 0.01^{dCD}	0.22 ± 0.01^{cB}
9.2	157 ± 4^{dAB}	0.12 ± 0.01^{cB}	27 ± 1^{cC}	0.31 ± 0.01^{cdC}	0.17± 0.01^{dC}	0.19 ± 0.01^{cC}
10.6	157 ± 9^{cdAB}	0.14 ± 0.01^{cAB}	30 ± 2^{bcB}	0.29 ± 0.01^{cD}	0.19± 0.01^{dB}	0.15 ± 0.02^{cD}
11.9	154 ± 7^{dAB}	0.15 ± 0.01^{cA}	33 ± 2^{dA}	0.27 ± 0.01^{eE}	0.22± 0.01^{cA}	0.12 ± 0.01^{cE}
Potato						
6.4	25 ± 1^{dB}	0.24 ± 0.01^{abA}	10 ± 1^{cB}	0.36 ± 0.01^{cdAB}	0.41± 0.01^{cA}	0.12 ± 0.01^{cB}
7.8	31 ± 1^{dB}	0.25 ± 0.01^{bA}	14 ± 1^{dB}	0.37 ± 0.01^{cAB}	0.43± 0.01^{cA}	0.12 ± 0.01^{eB}
9.2	44 ± 5^{eB}	0.25 ± 0.03^{bA}	18 ± 2^{dB}	0.37 ± 0.03^{bA}	0.41± 0.01^{cA}	0.13 ± 0.01^{eB}
10.6	64 ± 3^{cdB}	0.23 ± 0.01^{bAB}	25 ± 1^{cB}	0.36 ± 0.01^{bAB}	0.38± 0.01^{cA}	0.13 ± 0.01^{cAB}
11.9	260 ± 21^{cdA}	0.13 ± 0.01^{cB}	53 ± 2^{bA}	0.27 ± 0.01^{eB}	0.21± 0.01^{cB}	0.14 ± 0.01^{cA}
Tapioca						
6.4	14 ± 1^{deD}	0.25 ± 0.02^{aD}	7 ± 1^{dE}	0.40 ± 0.01^{bD}	0.48± 0.01^{abD}	0.14 ± 0.02^{cA}
7.8	16 ± 1^{eC}	0.26 ± 0.01^{bCD}	8 ± 1^{eD}	0.42 ± 0.01^{bC}	0.50± 0.01^{bC}	0.16 ± 0.01^{dA}
9.2	20 ± 1^{eB}	0.28 ± 0.01^{aBC}	10 ± 1^{eC}	0.43 ± 0.01^{aB}	0.52± 0.01^{bB}	0.15 ± 0.01^{dA}
10.6	21 ± 1^{dB}	0.30 ± 0.01^{aAB}	12 ± 1^{dB}	0.45 ± 0.01^{aA}	0.55± 0.01^{bA}	0.14 ± 0.01^{cA}
11.9	27 ± 1^{eA}	0.31 ± 0.01^{bA}	15 ± 1^{eA}	0.45 ± 0.01^{bA}	0.56± 0.01^{bA}	0.14 ± 0.01^{cA}

Conc. (%) = Concentration, expressed as % w/w gel; G'_1, G''_1 and $\tan(\delta)_1$ represent the elastic and viscous moduli, and the loss tangent at 1 Hz, respectively, obtained by fitting the data from the frequency sweeps to the power law model. The *a*, *b* and *c* values correspond to the exponents obtained from the fitting and quantify the dependence degree of dynamic moduli and loss tangent with the oscillation frequency. The presented data are the mean ± standard deviation. Different letters in each column indicate significant differences between means at $p < 0.05$. Lowercase letters compare samples at the same concentration, while capital letters compare samples of the same botanical origin.

Table S3. Deformation sweep values determined at 1 Hz.

Conc. (%)	Crossing Point Stress (Pa)	Crossing Point strain (%)	Max Stress (τ_{max}) (Pa)	Max Strain (%)
Wheat				
6.4	53 ± 5^{eE}	172 ± 15^{eC}	37 ± 2^{bE}	72.25 ± 0.01^{aC}
7.8	639 ± 19^{bD}	186 ± 1^{eC}	684 ± 37^{aD}	101.95 ± 0.08^{aB}
9.2	1553 ± 32^{aC}	216 ± 4^{cB}	1554 ± 32^{aC}	101.1 ± 0.1^{aB}
10.6	2176 ± 84^{aB}	233 ± 10^{cB}	2396 ± 144^{aB}	141.6 ± 0.3^{aA}
11.9	2770 ± 83^{aA}	270 ± 2^{cdA}	2847 ± 98^{aA}	140.55 ± 0.09^{aA}
Normal Maize				
6.4	212 ± 18^{cD}	165 ± 11^{eC}	148 ± 7^{aD}	51.85 ± 0.04^{bB}
7.8	711 ± 14^{aC}	195 ± 7^{eB}	583 ± 19^{bC}	72.37 ± 0.03^{bB}
9.2	1251 ± 66^{bB}	217 ± 7^{cAB}	1151 ± 43^{bB}	100.98 ± 0.07^{aA}
10.6	1454 ± 13^{bB}	241 ± 7^{cA}	1356 ± 58^{bB}	100.75 ± 0.01^{bA}
11.9	1867 ± 113^{bA}	244 ± 16^{cdA}	1687 ± 79^{bA}	100.8 ± 0.1^{bA}
Waxy maize				
6.4	26 ± 2^{fAB}	552 ± 41^{cA}	2 ± 1^{dC}	39 ± 5^{cA}
7.8	33 ± 7^{fAB}	534 ± 15^{cA}	3 ± 1^{dBC}	38 ± 1^{cA}
9.2	45 ± 2^{eA}	550 ± 36^{bA}	4 ± 1^{dB}	37.285 ± 0.001^{bA}
10.6	45 ± 2^{eA}	550 ± 36^{bA}	4 ± 1^{cB}	37.285 ± 0.001^{dA}
11.9	21 ± 3^{fB}	181 ± 22^{dB}	5 ± 1^{cA}	37.285 ± 0.002^{cA}
Normal rice				
6.4	104 ± 1^{dD}	325 ± 27^{dA}	33 ± 4^{bB}	37.327 ± 0.007^{cA}
7.8	147 ± 13^{dC}	273 ± 12^{dA}	59 ± 1^{cB}	37.344 ± 0.005^{cA}
9.2	215 ± 8^{dB}	258 ± 3^{bcA}	92 ± 3^{cAB}	37.339 ± 0.002^{bA}
10.6	256 ± 1^{dB}	265 ± 7^{cA}	148 ± 11^{cA}	51.97 ± 0.01^{cA}
11.9	345 ± 17^{eA}	285 ± 20^{cA}	94 ± 7^{cAB}	26.855 ± 0.003^{dA}
Waxy rice				
6.4	49 ± 2^{eE}	106 ± 5^{eE}	12 ± 1^{cB}	10.004 ± 0.001^{fC}
7.8	90 ± 1^{eD}	152 ± 2^{fD}	36 ± 1^{cdA}	26.856 ± 0.003^{dB}
9.2	120 ± 3^{deC}	187 ± 1^{cC}	38 ± 1^{cdA}	26.843 ± 0.001^{cB}
10.6	159 ± 9^{deB}	233 ± 9^{cB}	51 ± 3^{cA}	37.308 ± 0.004^{dA}
11.9	188 ± 6^{eA}	265 ± 8^{cdA}	51 ± 2^{cA}	37.301 ± 0.002^{cA}
Potato				
6.4	262 ± 2^{bD}	1687 ± 37^{bA}	5 ± 1^{cdB}	19.312 ± 0.001^{eA}
7.8	436 ± 22^{cD}	2254 ± 10^{bA}	6 ± 1^{dB}	19.315 ± 0.001^{eA}
9.2	650 ± 43^{cC}	2527 ± 292^{aA}	7 ± 1^{dB}	13.896 ± 0.001^{dB}
10.6	850 ± 9^{cB}	2686 ± 55^{aA}	9 ± 1^{cB}	13.898 ± 0.001^{fB}
11.9	1198 ± 85^{cA}	1415 ± 107^{bA}	36 ± 3^{cA}	13.899 ± 0.001^{fB}
Tapioca				
6.4	311 ± 2^{aE}	2286 ± 18^{aC}	4 ± 1^{dC}	26.839 ± 0.002^{dA}
7.8	446 ± 9^{cD}	2450 ± 19^{aBC}	5 ± 1^{dBC}	26.837 ± 0.001^{dA}
9.2	619 ± 9^{cC}	2535 ± 45^{aB}	6 ± 1^{dA}	26.840 ± 0.001^{cA}
10.6	718 ± 8^{cB}	2740 ± 106^{aA}	5 ± 1^{cBC}	19.314 ± 0.001^{eB}
11.9	919 ± 22^{dA}	2719 ± 16^{aA}	6 ± 1^{cAB}	19.312 ± 0.001^{eB}

Conc. (%) = Concentration, expressed as % w/w gel. The presented data are the mean ± standard deviation. Different letters in each column indicate significant differences between means at $p < 0.05$. Lowercase letters compare samples at the same concentration, while capital letters compare samples of the same botanical origin.
